# Monocyte Chemoattractant Protein‐1 and its role in Alzheimer’s Disease: A Longitudinal Study

**DOI:** 10.1002/alz70861_109030

**Published:** 2025-12-23

**Authors:** Jaipal Virdi, Barney A Schlinger, Daniel H Silverman

**Affiliations:** ^1^ University of California, Los Angeles, Los Angeles, CA USA

## Abstract

**Background:**

Neuroinflammation and systemic inflammation are increasingly studied in Alzheimer's disease (AD) pathogenesis. Monocyte Chemoattractant Protein‐1 (MCP‐1), a mediator of both processes, has demonstrated variable prognostic value depending on disease stage. To clarify its role in early AD, this study examines baseline MCP‐1 levels in cerebrospinal fluid (CSF) and plasma in individuals with mild cognitive impairment (MCI), assessing associations with FDG‐PET imaging outcomes over three years.

**Method:**

Imaging, demographic, and biomarker data were obtained from the Alzheimer’s Disease Neuroimaging Initiative for 97 individuals with MCI (MMSE = 27.4 ± 1.58; CDRSB = 1.54 ± 0.78), totaling 184 FDG‐PET scans at baseline and three‐year follow‐up. Plasma and CSF MCP‐1 levels were measured via multiplex assay with analyte‐specific quality control. Concentrations, originally pg/mL, were log‐transformed for statistical analyses. FDG‐PET scans were co‐registered and smoothed to 6 mm³. NeuroQ analysis involved scalp removal, rigid registration, and elastic transformation to generate 47 standardized VOIs (sVOIs) normalized to whole‐brain metabolism. A general linear model (GLM) assessed longitudinal sVOI changes, adjusting for age, gender, APOE4, and education.

**Result:**

Imaging analysis identified four sVOIs with significantly greater metabolic decline over three years associated with lower baseline plasma MCP‐1 levels. The GLM revealed that for each unit decrease in plasma MCP‐1, there was a corresponding reduction in metabolic decline: right posterior temporal cortex (3.3%, 0.033 ± 0.016, *p* = 0.04), right inferior lateral posterior temporal cortex (3.1%, 0.031 ± 0.014, *p* = 0.03), right superior lateral temporal cortex (3.9%, 0.039 ± 0.011, *p* = 0.0003), and right associative visual cortex (3.0%, 0.030 ± 0.013, *p* = 0.02). In contrast, higher CSF MCP‐1 levels were associated with more metabolism over the same period, with the right inferior lateral posterior temporal cortex exhibiting a 9.1% increase (0.091 ± 0.032, *p* = 0.007).

**Conclusion:**

Our findings show that lower plasma MCP‐1 levels are correlated to a significant decrease in metabolism in regions associated with AD over a three year period, while higher CSF MCP‐1 levels are correlated with an increase in metabolism.

